# Supplementary material for: Are carbon emissions trading and green financial instruments synergistic? -Comprehensive quantitative research based on content analysis
Source: PLoS One. 2024 Mar 7;19(3):e0298601. doi: 10.1371/journal.pone.0298601 (PMC10919601; doi:10.1371/journal.pone.0298601)
Supplement: S1 Data — (ZIP) [file pone.0298601.s001.zip › Data/地级市Z_F工作报告.zip!/╕ú╜¿/─╧╞╜╩╨/└┤╫╘╣1⁄2╓┌║┼í╛┬φ┐╦╩2╛▌═°í┐_2011.docx]

各位代表：

现在，我代表南平市人民政府向大会作政府工作报告，请各位代表连同《南平市国民经济和社会发展第十二个五年规划纲要（草案）》一并审议，并请市政 协各位委员和其他列席人员提出意见。

一、2010年和“十一五”时期经济社会发展回顾

2010年，在省委、省政府和市委的正确领导下，我市各级政府坚持以科学发展观为指导，全面贯彻党的十七大、十七届四中、五中全会和胡锦涛总书记等中央领导同志来闽考察重要讲话精神，按照省委八届九次、十次全会和市委三届八次、九次全会的部署，努力克服自然灾害的不利影响，经济社会发展取得新成效。全市生产总值728.7亿元，增长11.7%；财政总收入61.7亿元，增长18.2%，其中地方级财政收入38.6亿元，增长23%；规模以上工业产值777亿元，增长23.4%；农林牧渔业总产值262亿元，增长3.4%；全社会固定资产投资622亿元，增长23.9%；验资口径实际利用外商直接投资7079万美元，增长14.8%；外贸出口总额9.1亿美元，增长41%；社会消费品零售总额254亿元，增长16.3%；旅游接待总人数1319万人次，旅游总收入156亿元，分别增长11.2%、13.5%；居民消费价格总水平上涨4.2%；城镇居民人均可支配收入17332元，实际增长4.7%；农民人均纯收入6759元，实际增长6.1%；城镇登记失业率3.47%；人口自然增长率6‰。单位生产总值能耗降低3.5%，二氧化硫排放量下降0.75%；化学需氧量排放量减少0.95%。一年来的主要工作和成效是：

（一）全力抗灾救灾，恢复重建有力有效

去年我市先后遭受霜冻、冰雹、龙卷风等灾害，特别是6月中下旬特大暴雨洪灾持续时间长、范围广、强度大，地质灾害多发，造成严重损失。党中央、国务院和省委、省政府对我市抗灾救灾工作十分关心，给予有力指导和大力支持。灾情发生后，我市把确保人民群众生命安全放在第一位，精心组织，沉着应对，紧急动员一切力量抢险救灾，转移群众36.56万人，妥善安置受灾群众，努力把损失降到最低程度。灾后迅速展开恢复生产重建家园工作，成立了7个专项工作领导小组，将农村住房灾后重建作为重中之重，提升规划水平，创新运作机制，出台优惠政策，建立领导挂点、部门挂钩、干部驻点帮扶等制度，力争把灾后重建点建设成为新农村建设的示范点。根据省委、省政府的安排部署，厦门市积极开展对口援建工作，在人才、资金和技术等方面给予无私援助。经过努力，全市工农业生产和受损的基础设施基本恢复，10530户农村住房灾后重建户大部分于春节前搬入新居。

（二）突破发展瓶颈，基础设施建设加快

武邵高速公路、宁武高速公路枫坡至九曲互通段建成通车。宁武、龙浦、松建高速公路建设加快，京台高速公路南平段开工，邵光高速公路“初设”已获批复，延顺高速公路“初设”通过审查，南平绕城高速公路“工可”已获批复，顺邵高速公路完成“工可”编制。国省道干线公路网改造完成120公里。京福高速铁路和南三龙铁路南平段开工建设，向莆铁路南平连接线加快建设，浦建龙梅铁路、衢宁铁路南平段和南平东支线改造等项目前期工作扎实推进。电力设施进一步完善，建成220千伏电网主干线路33.5公里、35-110千伏电网线路140公里，220千伏变电站、110千伏变电站工程6项竣工、3项在建。

（三）奋力攻坚克难，“五大战役”成效明显

按照省委、省政府“大干150天，打好‘五大战役’”的决策部署，逐级逐项分解任务，明确责任，细化方案，强力推进工作落实。重点项目建设方面，实施重点项目229项，完成投资198.2亿元，增长38.4%，一批重大基础设施、重点产业和社会事业项目扎实推进，竣工投产和开工项目分别达95项、31项。新增长区域发展方面，武夷新区列入全省十大新增长区域之一，新区发展规划获得省上批准实施。成立新区管委会，出台扶持总部经济发展、产业组团招商引资和人才集聚的政策。完成城市总体规划大纲编制并向省上报批，开展核心区城市设计国际征集和评审工作，新区各专项规划编制同步开展。加快新区基础设施建设，303省道武夷山段拓宽改建工程加快建设，林后大桥、将口大桥、滨江西路、滨江东路等项目开工建设。城市建设方面，完成投资22.9亿元，新增城市绿地365公顷，新增和改造公园12座，更新公交车90辆，完成道路改造87公里，新建和改造公厕42座，景观整治、景区提升、立面再造、缆线下地等取得成效。组建城市管理行政执法局，违法违章建筑整治力度加大。顺昌污水处理厂和武夷山第二污水处理厂投入运行，建阳垃圾焚烧发电厂建设加快。小城镇改革发展方面，结合灾后重建、“造福工程”和中心村镇建设，推进2个省级试点镇规划建设，编制总体规划和专项规划，开展农村土地整治，实施城乡建设用地增减挂钩政策，稳步推进旧城镇、旧厂房、旧村庄改造。民生工程方面，实施57项社会事业和民生项目，为民办实事项目全面完成，十项事关群众利益工作有效落实。

（四）注重集中集聚，优势产业培育壮大

全社会工业增加值完成244.2亿元，二产比重比上年提高2.2个百分点。机械（装备）制造、冶金建材、林产加工、食品加工等产业产值均突破百亿元。全市产值超亿元企业145家、新增35家，其中超5亿元企业27家、新增8家。南铝高品质铝合金圆棒、太阳电缆500千伏超高压电缆生产线等48个重点项目建成投产。新兴产业加快培育，欧浦登液晶电视面板生产线正式投产，武夷国家级生物产业基地（南平）创建规划开始实施，武夷山大安源、顺昌华阳山通过国家4A级旅游景区验收，建阳胜德山庄、浦城丹桂大酒店通过国家四星级酒店评定，“印象大红袍”山水实景演出正式推出，武夷数字茶博馆等一批重点旅游项目相继建成。深化品牌带动，新认定中国驰名商标3件、福建省著名商标32件、福建名牌产品21个。松溪亚达通过融资型反向收购（APO）在美国上市融资，青松股份、元力股份在深交所创业板上市，全市上市企业增至7家。出台《关于进一步提升工业园区发展水平做强产业的实施意见》，闽北经济开发区、南平工业园区、荣华山产业组团持续拓展，园区基础设施建设完成投资48.4亿元，引进投资1000万元以上项目146项。各县（市、区）工业平台加快建设，一批工业项目落地投产。成功举办第十三届海峡西岸武夷国际投资洽谈会、海峡论坛·第三届武夷山朱子文化节、第四届海峡两岸茶业博览会暨武夷山茶节，积极参加“6·18”项目成果交易会、“9·8”投洽会、福建省与中央企业项目洽谈会等重大经贸活动，先后在台湾、厦门等地举办招商活动。

（五）持续强农惠农，现代农业稳步发展

全面落实各项强农惠农政策，发放粮食直补、良种补贴、农机具购置补贴、农资综合直补共3.5亿元，增长18%。粮食生产平稳增长，推广测土配方施肥315万亩，农业“五新”推广207项，农作物良种覆盖率96%。新建国家级农业标准化示范区5个。全市规模以上农产品加工企业710家、新增60家，农民专业合作社1007个、新增328个。灾后修复农田21万亩，实施土地开发整理7.8万亩，完成标准化农田建设、中低产田改造和高标准鱼塘改造4.4万亩，实施烟基项目受益农田13.3万亩，除险加固水库36座，新建生物防火林带1721公里。完成农村公路硬化375公里、农村公路安保工程630公里，新增农村饮水安全人口16.4万人，新建农村户用沼气池8140口。完成“造福工程”搬迁2.9万人。265个中心村完成规

划，189个中心村展开建设。新建农村商务信息服务站点676个。集体林权制度改革主体任务基本完成，配套改革积极推进。南平机制深化拓展，开展“千名农技干部下基层促增收”活动，健全农村“六大员”准入、考评和激励机制，137个市直单位挂钩帮扶118个村。

（六）坚持民生优先，社会建设扎实推进

全年新增城镇就业人员2.5万人，下岗失业人员再就业1.3万人，新增转移农村劳动力5.8万人。城镇职工基本医疗保险市级统筹启动实施，社保卡就诊一卡通发放工作有序推进。推进新农合扩面提标，参合率达98.2%。企业退休人员养老金水平继续提高，武夷山市新型农村社会养老保险试点顺利开展。实行城乡低保动态管理，城乡医疗救助制度进一步完善。建设各类保障性住房3633套，改造棚户区4203户。全面落实从义务教育到高等教育的各项助学优惠政策，6个县（市、区）实现“双高普九”，高中阶段毛入学率达82%。南平职业中专、闽北卫校新校区开工建设，闽北职业技术学院办学水平持续提升，武夷学院通过学士学位授予权评估。健全基层医疗卫生服务体系，实施基本药物制度改革，在全省率先实行公立基层医疗卫生机构药品销售零差率改革。持续稳定低生育水平，我市被列为全国人口和计划生育综合改革示范市，10个县（市、区）人口计生工作全面进入“国优”或“省优”先进行列。举办第三届科技成果交易会，签约项目54项，总投资8.6亿元。举办闽浙赣皖四省四市民间艺术节和南平市第四届农村文化艺术节，公共文化服务进一步加强。群众性精神文明创建活动不断深化。83%的建制村实现广播电视光缆联网，20户以上自然村广播电视“村村通”进展顺利，全面启动有线数字电视整体转换工作。群众体育活动蓬勃开展，参加第十四届省运会取得好成绩。严格落实节能减排和环保监管责任，重点流域水环境、重点行业和工业园区环境整治工作取得新进展，“四绿工程”有效推进。深入推进新一轮平安创建活动，开展校园安全专项整治行动，健全预防和处置医患纠纷的长效机制，全市信访态势总体平稳，人民群众对社会治安满意率和基本满意率达95.8%。强化安全生产管理，各类事故发生起数下降5%。妇女儿童、老龄和残疾人工作得到加强，社会福利和慈善事业稳步发展。援建四川省彭州市九尺镇任务圆满完成，援藏援疆工作扎实推进。民族宗教、气象防震、档案方志、老区库区等工作继续加强，国防动员、民兵预备役和双拥共建等工作取得新进展。

（七）推进依法行政，政府自身建设持续加强

各级政府自觉接受人大及其常委会监督，依法执行人大决定决议，认真办理人大代表议案和建议，积极配合人大常委会开展执法检查、人大代表视察及专项调研。支持人民政协履行政治协商、民主监督、参政议政职能，认真办理政协提案，积极采纳政协委员调研成果。全年办理市人大代表议案和建议136件、市政协委员提案271件，办复率均为100%。密切与各民主党派、工商联、各人民团体和无党派人士的联系，主动听取并积极采纳社会各界人士的意见和建议，推进决策的科学化、民主化、法制化。全面启动县、乡政府机构改革，市级政府机构改革进展顺利。行政监察不断加强，效能建设持续深化，行政自由裁量权进一步规范。审批制度改革扎实推进，政务公开有序推进。坚持政府部门述职述廉制度，严格落实廉洁自律各项规定，反腐倡廉工作不断加强。

各位代表，“十一五”期间，我们紧紧抓住海峡西岸经济区发展战略上升为国家战略的重大历史机遇，主动融入海西发展大局，持续拓展提升海西绿色腹地发展思路，经济社会发展迈上新台阶。五年来，综合实力不断增强，全市生产总值突破700亿元，实现了预期目标。与2005年相比，2010年全市生产总值、人均生产总值、财政总收入均翻一番，规模以上工业产值翻一番半，全社会固定资产投资翻两番。经济结构进一步优化，三次产业结构调整为21.9：41.9：36.2，二产比重上升7.3个百分点。基础设施明显改善，现代综合交通体系建设取得重大突破，11条高速公路列入国高网和海西网，通车里程420公里，在建里程500公里，均居全省前列。5条铁路列入新一轮海西铁路建设规划，3条高（快）速铁路开工建设，在建里程233公里。武夷山机场扩容改造和新机场规划建设工作取得新进展。南平首座500千伏变电站投入运行，电力网架结构从220千伏提升到500千伏。城市建设力度加大，城市功能进一步完善；新农村建设扎实推进，农村公共设施明显改善，城乡面貌日益改观。生态环境持续优化，生态市建设加快推进，国家级生态示范区和省级可持续发展实验区通过验收，生态环境质量保持在全省前列。资源保护力度进一步加大，全市木材年砍伐量从2005年的260万立方米调减到2010年的180万立方米，重点流域水环境综合整治取得实效。10个县（市、区）的12座污水处理厂全部建成投入运营，城市垃圾污水处理率不断提高。逐步淘汰高耗能、高污染的落后产能，实施一批示范性好、带动力强的资源综合利用项目，节能减排任务全面完成。民生水平稳步提高，大力发展社会事业，着力改善民生，城镇居民人均可支配收入、农民人均纯收入年均增长分别为11.9%、10.7%。城乡免费义务教育全面实现，公共文化服务体系初步形成，基本医疗保障制度覆盖全市居民，城乡低保实现应保尽保，老百姓得到更多实惠。

“十一五”发展所取得的成绩来之不易，这是省委、省政府和市委正确领导的结果，是全市人民凝心聚力、团结拼搏和各方面大力支持的结果。在此，我代表市人民政府，向为南平发展和海西绿色腹地建设付出辛勤劳动的全市人民，向给予政府工作大力支持和有效监督的人大代表、政协委员、各民主党派、工商联、各人民团体、无党派人士、离退休老同志和社会各界人士，向为南平发展作出贡献的中央、省属驻南平机构和闽北驻军、武警官兵、公安民警，向对我市灾后恢复重建给予无私援助的厦门人民，向关心支持南平发展的港澳台同胞、海外侨胞、国际友人，表示衷心的感谢！

在看到成绩的同时，我们也清醒认识到存在的问题。主要表现在：由于受特大暴雨洪灾的影响，去年个别经济指标未能完成年度预期目标；发展相对滞后，经济总量比较小，县域经济总体偏弱；二产比重较低，主导产业不突出，产业结构有待进一步优化；大项目、好项目不多，发展后劲还显不足；农民持续增收基础不牢靠，农田水利设施建设依然薄弱，抵御自然灾害能力不强；社会保障体系还不健全，社会事业发展和改善群众生活还有许多亟待解决的问题；节能减排和生态环境保护压力增大；安全生产和社会治安存在薄弱环节；城市管理问题仍然不少；政府职能有待进一步转变，一些干部思想不够解放、服务意识不强、办事效率不高，存在工作不落实等问题。我们要采取更加有力的措施，切实加以解决。

二、“十二五”时期发展目标任务

“十二五”是南平加快转变经济发展方式、推动跨越发展的关键时期，是建设海峡西岸经济区绿色腹地大有可为的重要机遇期。提请大会审议的《南平市国民经济和社会发展第十二个五年规划纲要（草案）》（以下简称《规划（草案）》），总结“十一五”规划的实施情况，分析“十二五”的宏观环境和形势，提出今后五年我市经济社会发展的目标任务和各项措施。

《规划（草案）》明确了我市“十二五”发展的指导思想是：高举中国特色社会主义伟大旗帜，坚持以邓小平理论和“三个代表”重要思想为指导，深入贯彻落实科学发展观，全面贯彻党的十七届五中全会、省委八届十次全会和市委三届九次全会精神，以科学发展为主题，以加快转变、跨越发展为主线，围绕“先行先试、加快转变、民生优先、党建科学”，统筹推进经济、政治、文化、社会、生态建设，充分发挥南平在海西纵深推进中的前锋作用，全力推动南平跨越发展，大力提升人民群众的幸福指数，为全面建成小康社会打下具有决定性意义的基础。

《规划（草案）》围绕主题、突出主线，明确了经济发展、民生发展、科教发展、可持续发展四大类40项主要目标，其中约束性14项、预期性26项，提出地区生产总值年均增长12%以上，年均增长速度赶超全省平均水平，到2015年力争全市地区生产总值达1400亿元以上，比2010年翻一番；财政总收入增速高于地区生产总值增速，达到123亿元，比2010年翻一番。这些发展目标体现质量、效益、结构、速度的统一，体现经济发展和改善民生的统一，体现先行先试与可行可为的统一，经过全市人民共同努力是可以实现的。

《规划（草案）》提出了今后五年经济社会发展的工作重点和主要任务，归纳起来有以下几个方面。一是建设竞争力强的现代产业支撑体系。大力支持现有企业增资扩产，积极引进大项目，提升做大传统产业，加快培育战略性新兴产业，增强产业园区集聚能力，建设海西先进制造业重要基地。加快构建大武夷旅游经济圈，全面提升旅游业发展水平，建设我国重要的自然和文化旅游中心。加快培育现代物流业和现代服务业。二是加快推进农业现代化和新农村建设。坚持城乡统筹发展，加大强农惠农力度，加快发展现代农业，改善农村生产生活条件，完善农村发展体制机制，夯实农业农村发展基础，拓宽农民增收渠道，提高农业产业化和农民生活水平。三是进一步完善现代基础设施体系。加快建设以高（快）速铁路、高速公路为骨架的现代综合交通体系，形成“南接北联”和“西进东出”的海西区域交通枢纽。优化能源保障结构，完善电网配套设施。构建与经济社会发展相适应的防灾减灾体系，提升城乡防灾减灾能力。四是加快武夷新区开发建设步伐。高起点、高标准推进开发建设，完善新区规划体系，全面展开基础设施建设，着力引进一批产业项目，形成初具规模的总部经济，打造先进制造业和新兴产业基地。加强生态建设与文化保护，创新开发体制机制，建设全省先行先试示范区。五是加快城乡区域一体化发展。落实主体功能区战略，统筹推进城乡发展，加快城镇化进程。加大南平中心城市建设力度，提升县域城市功能，增强县域城市承载能力。加快推进小城镇和中心村镇建设，促进城乡规划、基础设施建设、基本公共服务一体化。六是积极落实扩大内需战略。保持投资较快增长，优化投资结构，改善投资环境，强化投资支撑带动作用。扩大和活跃消费需求，稳步提高公共财政用于改善民生投入的比重，提高城乡居民消费能力。健全和完善内需持续稳定增长的政策措施，培育消费市场，构建扩大内需的长效机制。七是持续加强生态文明建设。强化生态保护，加大节能减排攻坚力度，发展循环经济和低碳产业，加快建设生态市，把生态优势转化为产业优势，增强可持续发展能力。八是优先保障和改善民生。积极扩大就业促进创业，完善城乡社会保障体系。加强城乡公共卫生服务，大力发展文化体育事业，提高人民群众健康水平和文明素质。加强社会管理和民主法治建设，保持社会和谐稳定。九是实施科教兴市和人才强市战略。优先发展教育事业，加快完善覆盖城乡的基本公共教育服务体系。着力推进科技进步和创新，加快科技成果转化与应用。加强人才队伍建设，完善人才激励保障机制，促进人才集聚，努力建设人才强市。十是深化改革和扩大开放。深化行政管理体制改革，稳步推进财政金融体制改革，增强市场主体的动力和活力。提高利用外资水平，转变外贸增长方式。构建对台交流合作平台，加强与港澳经贸合作，扩大区域经济协作，全面提升对外开放水平。

三、扎实做好2011年工作

今年是“十二五”开局之年。我们要全面贯彻落实省委、省政府和市委的部署，坚持以科学发展为主题，以加快转变、跨越发展为主线，继续打好“五大战役”，大干“十二五”开局之年，推动科学发展、跨越发展迈出坚实步伐。经济社会发展的主要预期目标是：地区生产总值增长13%；财政总收入和地方级财政收入增长15%，其中市本级财政总收入增长15%，地方级财政收入增长20%；规模以上工业产值增长25%；农林牧渔业总产值增长6%；全社会固定资产投资增长22%；验资口径实际利用外商直接投资增长10%，实际利用内资增长10%，外贸出口总额增长15%；旅游接待人数和收入分别增长12%和15%；社会消费品零售总额增长15%；居民消费价格总水平涨幅控制在4%左右；城镇居民人均可支配收入增长10%，农民人均纯收入增长10%；人口自然增长率控制在7‰以内；城镇登记失业率控制在3.5%以内；完成单位生产总值能耗、化学需氧量、氨氮、二氧化硫、氮氧化物节能减排任务。

实现上述目标，必须着重做好以下八个方面工作：

（一）全力打好“五大战役”，确保开好局起好步

打好重点项目建设战役。坚持先谋后动、动则必快、动则必成，抓紧谋划实施一批大项目、好项目，做到新增一批、储备一批、开工一批、投产一批。全年实施重点项目230项，其中在建重点项目178项、预备重点项目52项，总投资超过1500亿元，年度计划投资超过200亿元。产业方面，加快实施南铝板带三期、光泽圣农产业化、青松股份松节油深加工、邵武武夷烟叶打叶复烤生产线等79个在建工业项目。推进武夷山大红袍山庄、建阳印象·考亭葡萄主题公园、邵武云灵山、顺昌宝山4A级旅游景区和浦城五星级酒店等23个旅游项目建设。实施闽北（光泽）物流商贸城、建瓯仓储物流中心等18个商贸服务项目。交通、能源方面，实施铁路项目6项、高速公路项目8项和武夷山机场改扩建项目。竣工投产一批输变电工程，实施一批农网改造项目。农业方面，建设一春（延平）农业生态科技园、顺昌无患子生物原料林基地等9个重点项目。

打好新增长区域发展战役。全面推进武夷新区开发建设，全年计划完成投资175亿元以上。规划建设方面，完成《武夷新区城市总体规划》成果编制和评审报批工作，继续推进新区各专项规划、核心区控制性详细规划的编制、评审和报批工作。推进新区京福高铁、宁武高速、芹口交通枢纽和站前广场等重大基础设施建设，加快城市快速通道及景观带建设。开工建设城市规划展示馆、青少年活动中心等一批项目。提升闽北经济开发区集聚能力，促成雨润食品、武夷兴华实业、派森控股等一批项目开工建设，争取新引进项目30个以上。经营运作方面，探索多渠道融资模式，加强与农发行、国开行、农行、兴行等金融机构的战略合作，运用BOT、BT等融资手段，进一步拓展融资平台。加快推进与跨国公司的战略合作，吸引中央、省属企业和大型民营企业到新区设立地区总部、研发中心、采购中心。加强政策研究，用足用好中央苏区、革命老区优惠政策，在用地、产业、金融、财税、人才、对台等方面，积极向上争取更多的政策、资金和项目支持。保护控制方面，强化武夷山“双世遗”保护，严把项目审批关，进一步加大环境综合整治力度，努力遏制抢建抢盖、占地圈地、乱垦茶山等各类违法违规行为，提升环境质量。

打好城市建设战役。做好城市专项规划编制，加强重要区域、重点地段设计，各县（市、区）在9月底前全面完成新一轮城市总体规划报批和备案。强化市容市貌整治，提高城市精细化管理水平。加强城市道路建设，各县（市、区）至少完成一条城市主次干道路面“白改黑”，实施一批既有道路照明系统改造项目，实现城区主干道架空缆线全部下地，建成一批公共停车场。推进城市立体绿化和公园建设，做到步行10-15分钟就能看到绿地，各县（市、区）建成一座面积6公顷以上的城市综合性公园。加快供水、供气、排水设施建设，确保城市居民饮用水达到国家卫生标准。建成一批城市垃圾处理项目，完成建阳垃圾焚烧发电厂主体工程建设，加快城市污水处理厂配套管网铺设进度，力争污水处理率超过80%。全市上半年建成38座公厕。创建一批物业管理示范小区，建设一批宜居城市综合体。

打好小城镇改革发展战役。推进建瓯徐墩、浦城仙阳、邵武和平、建阳水吉等4个省级和9个市级小城镇综合改革试点建设，加快规划编制，全面推进基础设施、市政公用事业和景观配套建设，促进产业和人口集聚，建设“功能齐备、设施完善、生活便利、环境优美、保障一体”的宜居城镇综合体。加快推进中心村镇规划建设，因地制宜发展一批各具特色的中心镇、中心村，促进农村居住社区化、就业多元化、保障社会化、公共服务一体化。

打好民生工程战役。继续集中财力办好惠民利民实事，加快推进一批重大社会事业项目建设，着力解决人民群众关心的热点难点问题，让人民群众生活得更方便、更安稳、更有质量。就业和社会保障方面，实现新型农村社会养老保险制度全覆盖；建设各类保障性住房1.39万套、83.4万平方米，实施各类棚户区改造5387户、43.1万平方米；开工建设一批县级社会福利中心、农村敬老院和社区居家养老服务中心；实施特殊教育提升工程、残疾人托养、残疾儿童康复项目；开展“光明行动”，实施1000例贫困白内障患者复明手术；扶持妇女、残疾人、计生家庭创业就业。医疗卫生方面，推进县、乡、村三级医疗机构标准化建设，提高新农合和城镇居民基本医疗保险政府补助标准，为城乡居民提供10项公共卫生免费服务项目，实施城乡困难居民和儿童特殊病种医疗救助，推进免费孕前优生健康检查试点，开展农村孕妇、城市低保孕妇免费产前筛查诊断，提高农村部分计生家庭奖励扶助和计生家庭特别扶助标准。教育文化方面，落实中职教育优惠政策，实施中小学校舍安全工程项目68个、10.5万平方米，加快幼儿园发展，实施城乡低保家庭入园幼儿保教费补助，完善普通高中学生国家助学金制度，提高高等学校国家助学金标准，解决进城务工人员随迁子女就学；实施农家书屋建设和有线广播“村村响”工程，加快乡镇综合文化站和城区影院建设，推进县（市、区）妇女儿童活动中心和乡镇农民体育健身活动中心建设。强农惠农方面，调整完善政策性农业保险，建设村级综合服务场所，开展农村“村村通客车”工程，建设农村社区综合维修服务体系，开展农村实用技术远程培训，继续实施“造福工程”搬迁农村贫困群众1万名。便民生活方面，抓好主要农产品、基本生活必需品、重要生产资料的生产、供应和价格监管，进一步加强“菜篮子”工程建设，新建城市副食品基地30个，蔬菜基地种植面积扩大到5万亩；加快城乡便民商业网点建设，新建社区便利店50个，新增“万村千乡市场工程”农家店135家，新建和升级标准化农贸市场15个；提升公交服务，推进城乡绿化和保洁工作，实施城乡通信信息化提升工程。和谐平安方面，治理“餐桌污染”、建设食品放心工程，建设自然灾害避灾点，深化平安创建，支持闽北驻军改善生产生活和科技拥军、文化拥军项目建设。防灾减灾方面，加快建设气象灾害和动植物疫病防控体系，增强重大灾害预警预报、快速反应和紧急救援能力。开展重大地质灾害点治理和山洪灾害防治，努力消除灾害隐患。

（二）加速产业转型升级，调整优化经济结构

着力壮大产业群。组织实施铝加工、竹加工、肉鸡加工、纺织服装、电线电缆等五个百亿产业链规划，有针对性地策划生成一批项目，延伸产业链，做大做强传统优势产业。着力培植和引进一批龙头企业，重点抓好南纸竹纤浆粕技改、雨润集团肉联加工等百个投资5000万元以上的产业项目，积极支持现有企业增资扩产，增强新产品开发和品牌创建能力。发展生物（医药）、光电、电子信息、现代服务业、新材料、新能源、节能环保等战略性新兴产业，强化政策扶持、人才培养、技术研发等配套服务，加快新兴产业的培育形成。实施年新增产值1000万元以上的新增长点项目138个，力争全市工业总产值超1000亿元。积极推进工业园区整合提升，鼓励市本级三大产业组团及一批县级园区扩区升级，引导产业链关联企业向园区集聚发展。

加快发展大武夷旅游经济。加大旅游产品和市场开发力度，重点在做大规模、培育特色、塑造品牌上下功夫，全面提升大武夷旅游的知名度和影响力。依托武夷山“双世遗”品牌，整合全市旅游资源，丰富旅游产品，拓展旅游线路，有针对性地开展宣传推介，加强与周边地区旅游协作，加快培育大武夷“旅游套餐”。着力引进大型旅游龙头企业，建设精品项目，形成一批有特色、有规模的旅游综合体，促进旅游深度开发，延伸产业链，延长游客停留时间。按照建设武夷山国际性旅游度假城市的要求，完善旅游基础设施和配套服务，提高接待能力。积极推进一批旅游景区和特色乡村旅游项目建设，争创一批国家A级旅游景区和星级乡村旅游经营单位，提升景区产品的档次和品位。加强旅游市场管理，推进诚信建设，提升服务质量。

大力发展现代服务业。积极培育现代物流业，开工建设10个物流项目，升级改造10个专业批发市场。完善武夷山“陆地港”的报关、报验等口岸服务功能，建设综合性物流中心和公共物流平台。推进城市社区和农村商业流通网络建设，为城乡居民扩大消费创造条件。继续实施家电下乡和以旧换新政策。发展以社区服务、养老服务和病患陪护为重点的家庭服务业。扶持武夷山会展业发展，完善会展设施和服务网络。

（三）加快基础设施建设，不断改善发展条件

加快建设现代立体交通。全力推进松建、龙浦、宁武和京台高速公路南平段建设，开工建设邵光、延顺高速公路，做好南平绕城、顺邵高速公路前期工作，确保龙浦高速公路建成通车。加快建设京福高速铁路、南三龙铁路南平段和向莆铁路南平连接线。开工建设浦建龙梅铁路、衢宁铁路南平段。继续推进既有铁路改造前期工作。加快建设省道303线武夷山枫坡至机场公路，省道202线、302线政和段等省道干线改造项目。推进武夷山机场扩能升级，做好新机场建设前期工作。

强化电力能源保障。进一步完善220千伏主干电网，建成220千伏线路280公里、35-110千伏线路211公里，新建、扩建220千伏变电站2座，新建、扩建110千伏变电站11座。开展新一轮农网改造升级，继续做好邵武火电厂扩建项目前期工作，积极推进闽北第二个500千伏输变电项目、南平核电项目前期工作。

（四）积极发展现代农业，夯实农业农村基础

提高农业产业化水平。稳定发展粮食生产，提高农业综合生产能力，保障农产品供给安全。用工业化理念谋划农业发展，促进优势特色农产品集约化、标准化、机械化生产，加快农业产业化进程。鼓励扶持龙头企业建立带动力强、辐射面广的特色农产品生产基地，促进农产品就地深加工，提高附加值，全市新增规模以上农产品加工龙头企业30家。提高农村生产经营组织化程度，新增农民专业合作社200个。大力发展品牌农业，推进无公害、绿色和有机农产品认证，加强农产品注册商标、地理标志产品和地理标志证明商标申报、保护和使用。完善农业科技服务体系，推广农业“五新”230项。健全农产品质量安全检验检测体系，进一步加强农产品质量安全监管。

拓宽农民增收渠道。千方百计增加农民收入，努力实现农民收入增长与城镇居民收入增长基本同步。大力发展笋竹、茶叶、烤烟、蔬菜、食用菌等特色优势经济作物，发展畜牧水产规模化健康养殖，推进油茶、桂花、无患子、油桐等林业特色产品的种植和开发，提高农民种养收益。推广“龙头企业+合作组织+农户”经营模式，发展订单农业。培训农民10万人次以上，促进农村劳动力就地就近转移就业，增加农民务工收入。落实各项强农惠农政策，确保各项政策补贴、项目补助及时足额到位。认真组织实施扶贫开发政策，对贫困农户帮扶到户。

推进农田水利等基础设施建设。抓住中央加大水利建设投入的有利时机，深化水利改革，发展民生水利。策划生成并启动实施一批防洪排涝和水利基础设施项目，改善农村生产生活条件。加快冬春水利建设和水毁工程修复，汛期前基本修复因灾损毁的水利工程，完成30座小（一）型重点水库除险加固工程。继续实施闽江上游建溪防洪三期南平段、富屯溪防洪三期邵武段、顺昌段工程，开工建设江南新区、武夷新区防洪工程，推进光泽茶富等大中型水库项目前期工作。解决15万人饮水安全问题。开展重点地区中小河流治理，完成绿色屏障水土流失治理2.5万亩，新增节水灌溉面积5万亩。实施农业综合开发，完成中低产田改造、小流域治理、高标准农田建设示范工程项目7万亩。完成土地整理3.5万亩，补充耕地1.7万亩，继续改善烟田基础设施。推广测土配方施肥，提高耕地质量。推广先进实用的农机具和农机化新技术，提高主要农作物耕、种、收等生产环节的机械化水平。继续实施竹山引水灌溉示范项目，新建竹山机耕路1000公里、生物防火林带300公里。实施“情系三农惠万家”移动信息化工程。硬化农村公路300公里。建设沼气池8000口。实施农村改厕项目1万个。

深化农村改革创新。在稳定和完善农村土地承包关系的基础上，逐步推行农业用地依法、自愿、有偿、有序、合理流转，鼓励农业种植大户发展规模经营。深化集体林权制度改革，加快林权证发放，扩大林权抵押贷款，推进采伐管理制度改革。完善农村金融服务，扩大农业保险范围，引导金融机构增加农业贷款。持续深化“南平机制”，选派第八批科技特派员驻村开展科技服务，继续开展“挂村服务，互动联动”工作。加强乡镇“三农”服务中心和村级综合服务站建设，发挥农村“六大员”队伍的作用，提升服务能力。

（五）增强城市集聚功能，推动区域协调发展

加快南平中心城市建设。进一步提升城市规划水平，优化功能布局，完善配套设施，建设组团式山水园林宜居城市。抓好安济、大家厂、西门岭等片区规划建设，严格控制建筑密度，降低开发强度，留足公共空间，做好绿化和景观配套，建设现代精品小区。加快沿江快速通道、闽江大桥和滨江东路建设，建成剑州大桥、常坑大桥，新建创世纪、紫云社区等停车场，改善城市综合交通环境。实施明翠公园一期工程、剑州大桥和玉屏桥头绿化工程等一批城市景观、重要节点改造项目，推进街巷保洁、广告清理、立面再造和夜景工程，实现城市“净化、绿化、美化、亮化”，改善人居环境。加强城市综合执法，大力整治违法违章建筑，提高城市综合管理水平。

提升县域城市功能。继续策划实施一批基础性、功能性、产业性项目，引导产业和人口向县域集聚，增强县域城市承载能力，加快县域经济发展。邵武市加快建设海峡西岸经济强市和闽赣周边有影响的区域性中心城市，增强辐射效应；武夷山市、建阳市加快与武夷新区的规划对接，实现功能互补、联动发展；建瓯市、浦城县、顺昌县在城市规模扩张和集聚上下功夫；光泽县、松溪县、政和县进一步优化城市功能布局，培育各具特色、充满活力的县域经济体。

统筹推进区域发展。加强与沿海发达地区经贸协作，建立更加紧密的山海协作、区域协作长效机制。进一步落实国家和省上各项扶持政策，建立项目跟踪、服务协调机制，抓好协作项目的落实。实施老区村跨越发展工程，整村推进扶贫开发，改善原中央苏区、革命老区、民族地区、偏远山区、水库库区生产生活条件，增强自我发展能力。

（六）加大改革开放力度，增强经济发展活力

大力开展招商引资。围绕闽北产业布局和发展方向，突出工业、旅游和新兴产业等重点，精心策划生成一批关联性强、上下游配套的项目对外招商，主动承接发达地区符合环保要求的产业转移。建立健全招商引资奖励和考核机制，做细做实项目前期工作，精心编制招商项目册、服务指南和优惠政策，对一些重大项目和捆绑项目实行“一事一议”、“一企一策”，提高项目洽谈成功率。采取以商招商、上门招商、网络招商，鼓励闽北籍客商“回归”等方式，拓宽招商渠道。精心办好“5·13”投洽会和海峡两岸茶业博览会，做好赴港澳经贸招商活动，积极参加“6·18”项目成果交易会、“9·8”投洽会以及我省在北京举办的大型民营企业产业项目洽谈会等重大展会活动，进一步加强与中央企业、省属企业、大型民营企业和跨国公司的对接和洽谈，力求在引进大项目上取得新突破。

推进重点领域改革。进一步完善国有资产监管体系和运营格局，做大做强市属投融资平台。成立市土地收储总公司，完善土地管理、收储、运营的体制机制。继续深化金融改革，推进政银企合作，鼓励更多的金融机构在南平设立分支机构，组建南平农村商业银行，培育发展村镇银行，推进小额货款公司试点工作，加强中小企业信用担保体系建设，规范融资性担保公司发展。加快重点上市后备企业培育工作，推进企业上市融资，力争企业发行债券取得新进展。创新人才工作机制，组织实施人才政策，积极稳妥推进人事制度和收入分配制度改革，留住人才、吸引人才、用好人才。扩大对外交流合作。拓展服务周边地区发展的对外开放综合通道，更加广泛地参与区域分工和协作。以海峡两岸经济合作框架协议正式实施为契机，深入推进南台经贸文化交流合作，加强与国外、港澳台地区经济技术交流合作。积极调整外贸结构，推动出口基地建设，培植出口大户，提高利用外资水

平。鼓励扶持企业参加各类展会，拓展国际市场。

（七）持续推进生态建设，保护闽北青山绿水

保护生态资源。加强森林资源保护，继续控制木材采伐量，严格限制新上木材加工企业。大力开展造林绿化，推进城乡一体化“四绿工程”，完成植树造林118万亩。建设一批城市片林和森林公园，扩大绿地面积。调整优化树种林种结构，实施洪灾滑坡林地治理，增强森林涵养水源能力。加强水资源开发利用管理，强化河岸生态地保护，严格河道采砂监管。加大矿产资源保护力度，规范勘查开发行为。

抓好环境整治。深入推进重点流域水环境综合整治，进一步加强饮用水源保护区内各类污染源的排查清理，确保饮水安全。大力开展工业园区和重点企业污染整治，实施造纸、制药等工业污染深度治理，推进畜禽养殖污染治理，严格控制重点流域网箱养殖。继续开展农村环境连片整治，深入实施“三清”示范工程，积极创建生态乡镇、生态村。

推进节能减排。认真实施生态功能区划，加强重点区域环境影响评价，落实环保“三同时”。严格节能减排责任，清理整合竹木加工、矿产加工、建材等资源消耗型企业，加快淘汰水泥、造纸、皮革、冶炼等落后产能。严把新上项目产业政策、资源消耗和环境保护审批关，推动新技术、新装备的研发与应用。推进工业园区污水处理项目建设，促进企业污染物稳定达标排放，建设生态工业园区。严格执行行业排放标准，推行清洁生产审核，力争重点行业、重点企业能耗水平明显下降。大力发展循环经济、清洁生产，力争创建一批省级以上循环经济示范园区和示范企业。

（八）着力保障改善民生，提高人民群众幸福指数

坚持教育优先发展。大力推进“双高普九”，进一步调整城乡中小学布局，实施义务教育学校标准化建设，促进城乡教育资源均衡配置，加快普及高中阶段教育。完成南平四中校园改造工程，南平一中江南校区投入使用。做好浦城一中、武夷山一中、松溪一中创建“省一级达标校”工作。提升职业院校办学水平，加快培养服务闽北发展的实用型、技能型人才。建成南平职业中专、南平卫校一期工程并投入使用。支持武夷学院提高办学水平。积极发展学前教育，合理规划布局公办幼儿园，积极扶持、规范管理民办幼儿园。加强师德建设，提高教师队伍素质。支持民办教育，扶持发展特殊教育和民族教育。大力发展继续教育，加快发展老年教育，构建终身教育体系。

提高医疗卫生服务水平。扎实推进医疗卫生体制改革，全面加强医疗机构基础设施建设，扩充医疗资源总量，争取千人床位数和医生数达到全省平均水平，推动公共卫生服务均等化。进一步推进基本药物制度改革和基本医疗保障改革。加快推进社保卡就诊一卡通、健康档案系统管理等医疗卫生信息化建设。加强疾病预防控制和县乡卫生监督体系建设，强化妇幼卫生、精神卫生工作。深化人口和计划生育综合改革，持续稳定低生育水平，提高出生人口素质，武夷山市、浦城县力争创建国家级计生优质服务先进单位。

加快科技文化事业发展。强化科技创新平台建设，加强产业关键技术攻关，组织实施一批重点科技项目，加速科技成果转化应用。加大政府公益性文化事业的投入，加快城乡公共文化设施建设，力争村俱乐部覆盖率达90%，推进数字电视整体转换。大力宣传闽北历史文化，重视发掘保护文化遗产，推进文化体制改革，加强文化市场和互联网管理。加快城乡公共体育设施建设，广泛开展群众性体育活动，提升竞技体育水平。落实民族政策，依法管理宗教事务。

做好就业和社会保障工作。落实就业再就业政策，新增城镇就业2.3万人，转移农村劳动力5.5万人。继续开展公共就业服务专项活动，重点做好高校毕业生、城镇就业困难人员、农村转移劳动力的就业工作。改善就业创业服务，强化职业技能培训，完善小额贷款和社保补贴政策，提高城乡劳动者创业就业能力。扩大养老保险覆盖面，提高企业离退休人员养老金水平，推进机关和事业单位养老保险制度改革，完善被征地农民养老保障制度。健全城乡医疗救助制度，加强城乡困难家庭专项救助和临时救助，提高农村五保供养水平。实施新一轮妇女儿童发展纲要，维护妇女儿童合法权益。改善优抚对象和革命五老人员待遇，发展社会福利、慈善和残疾人事业。

维护社会和谐稳定。全面提升社会管理服务水平，健全基层管理和服务体系，提高城乡社区自治和服务功能。完善信访工作机制，畅通群众诉求表达、利益协调、权益保障渠道，从源头上提升预防和化解矛盾纠纷能力。深化“平安南平”建设，加强基层普法教育，健全社会治安防控体系。加强生产安全、交通安全、校园安全、消防安全、社会安全的监督管理，健全突发公共事件预警和应急处置机制。强化国防教育、国防动员及国防后备力量建设，争创新一届双拥模范城市。

四、加强政府自身建设

各级政府要始终坚持执政为民，勤政廉政，优化服务，努力建设人民满意的政府。

（一）加强学习，提高科学发展能力。深入学习实践科学发展观，进一步解放思想、更新观念，提高科学决策、统筹协调能力。加强公务员教育培训，不断提高学习力，进而转化为推动南平科学发展、跨越发展的思路、决策、能力和本领。学习借鉴兄弟地市的好经验、好做法，打破思维定势，把握发展机遇，创新体制机制，提高运作能力，争取更大作为。

（二）转变职能，提高公共服务能力。坚持依法行政，自觉接受人大及其常委会的法律监督、工作监督，主动接受政协的民主监督，虚心接受社会舆论和人民群众的监督，提高政府决策科学化、民主化水平。持续推进政府机构改革，稳步推进事业单位分类改革，强化社会管理和公共服务。全面推进政务公开，深化行政审批改革，完善政务信息平台、行政服务中心和招投标中心，简化审批程序，提高行政效率，打造统一、公平、透明、可预见的政策环境，廉洁、高效的行政环境，安全、舒适、整洁的生活环境。

（三）强化责任，提高决策执行能力。增强“等不起”、“慢不得”、“坐不住”的紧迫感、危机感和责任感，以“5+2”、“白加黑”的拼搏精神投入工作，形成比学赶超、创先争优的良好氛围。大力治庸治懒，改进工作作风，加强效能督查，严格实行问责，确保政令畅通，实现政府工作全面提速。加强对重点储备项目前期、重点项目建设、工业（含商贸物流、旅游）在建项目、招商引资在谈项目、征地拆迁等重点工作的考核考评，确保各项工作不折不扣落实到位。

（四）从严治政，提高廉洁自律能力。深化政府系统反腐倡廉工作，建立健全教育、制度、监督并重的惩治和预防腐败体系，从源头上防治腐败。进一步加强廉政制度建设，规范行政行为，减少自由裁量权。强化审计监督和行政监察，加大对建设工程项目招投标、经营性土地使用权出让、产权交易等重点领域和关键环节的监督，坚决纠正损害群众利益的不正之风，树立为民、务实、清廉的政府形象。

各位代表，实现南平“十二五”规划目标，使命光荣，责任重大。让我们紧密团结在以胡锦涛同志为总书记的党中央周围，深入贯彻落实科学发展观，在中共南平市委领导下，凝聚全市广大干部群众的智慧和力量，坚定信心，开拓创新，拼搏奋进，以优异的成绩迎接中国共产党成立90周年！
